# Poor Prognosis among Radiation-Associated Bladder Cancer Is Defined by Clinicogenomic Features

**DOI:** 10.1158/2767-9764.CRC-24-0352

**Published:** 2024-09-04

**Authors:** N. Ari Wijetunga, Kathryn H. Gessner, Krishna Kanchi, Jay A. Moore, Zoe Fleischmann, Dexter X. Jin, Garrett M. Frampton, Michael Sturdivant, Michael Repka, Shivani Sud, David L. Corcoran, Matthew D. Galsky, Matthew I. Milowsky, Sara E. Wobker, William Y. Kim, Tracy L. Rose, Jeffrey S. Damrauer

**Affiliations:** 1 Radiation Oncology, University of North Carolina, Chapel Hill, North Carolina.; 2 Department of Urology, University of North Carolina, Chapel Hill, North Carolina.; 3 Lineberger Comprehensive Cancer Center, University of North Carolina, Chapel Hill, North Carolina.; 4 Genetics, University of North Carolina, Chapel Hill, North Carolina.; 5 Foundation Medicine, Inc., Boston, Massachusetts.; 6 Department of Pharmacology, University of North Carolina at Chapel Hill, Chapel Hill, North Carolina.; 7 Division of Hematology and Medical Oncology, Icahn School of Medicine at Mount Sinai, New York, New York.; 8 Department of Medicine, Oncology, University of North Carolina, Chapel Hill, North Carolina.; 9 Pathology and Lab Medicine, University of North Carolina, Chapel Hill, North Carolina.

## Abstract

Radiotherapy (RT) for prostate cancer has been associated with an increased risk for the development of bladder cancer. We aimed to integrate clinical and genomic data to better understand the development of RT-associated bladder cancer. A retrospective analysis was performed to identify control patients (CTRL; *n* = 41) and patients with RT-associated bladder cancer (*n* = 41). RT- and CTRL-specific features were then identified through integration and analysis of the genomic sequencing data and clinical variables. RT-associated bladder tumors were significantly enriched for alterations in *KDM6A* and *ATM*, whereas CTRL tumors were enriched for *CDKN2A* mutation. Globally, there were an increased number of variants within RT tumors, albeit at a lower variant allele frequency. Mutational signature analysis revealed three predominate motif patterns, with similarity to SBS2/13 (APOBEC3A), SBS5 (ERCC2/smoking), and SBS6/15 (MMR). Poor prognostic factors in the RT cohort include a short tumor latency, smoking status, the presence of the smoking and X-ray therapy mutational signatures, and *CDKN2A* copy number loss. Based on the clinical and genomic findings, we suggest at least two potential pathways leading to RT-associated bladder cancer: The first occurs in the setting of field cancerization related to smoking or preexisting genetic alterations and leads to the development of more aggressive bladder tumors, and the second involves RT initiating the oncogenic process in otherwise healthy urothelium, leading to a longer latency and less aggressive disease.

**Significance:**

Clinicogenomic analysis of radiation-associated bladder cancer uncovered mutational signatures that, in addition to a short tumor latency, smoking, and *CDKN2A* loss, are associated with a poor outcome. These clinical and genomic features provide a potential method to identify patients with prostate cancer who are at an increased risk for the development of aggressive bladder cancer following prostate RT.

## Introduction

The development of bladder cancer following pelvic radiotherapy (RT) for prostate cancer is an increasingly recognized phenomenon (1–4). More than half of patients with intermediate or high-risk prostate cancer initially undergo RT, representing nearly 150,000 patients per year (5–7). In total, 1% to 2% of patients with prostate cancer develop subsequent bladder cancer, and patients who receive RT as primary treatment for prostate cancer are 1.5 to 3 times more likely to develop bladder cancer than those who received radical prostatectomy (refs. 3, 8–11). The risk of bladder cancer increases as the duration of time since RT increases, with a majority of cases occurring more than 5 or 10 years after RT (12, 13). RT-associated bladder cancer also has a higher stage and grade at initial diagnosis than *de novo* bladder cancer, suggesting that the molecular underpinnings of these entities may be distinct (14–17).

Numerous studies have shown a relationship among ionizing radiation, urothelial dysplasia, and DNA damage (18–20). The predilection to tumorigenesis after radiation exposure varies with the type and dose of radiation, organ studied, and patient characteristics, which determine the likelihood of DNA damage and the DNA repair capacity of radiation-exposed cells (18, 21). Samples of bladder epithelium from people living in radio-contaminated areas near the Chernobyl accident demonstrated a high frequency of *TP53* mutations and a high frequency of specific single bp substitutions (19, 20). Such mutational patterns and RT-induced field defects may increase an individual’s risk for bladder cancer development while imprinting a distinct molecular signature characteristic of RT-associated bladder cancer. For example, prior data have shown an increased number of balanced inversions in RT-associated cancers (22). Additionally, the only other study, to our knowledge, that genomically characterized radiation-specific bladder cancer noted an increased number of short insertions and deletions (indels) compared with an independent control (CTRL) cohort (17).

Here, we report the clinicogenomic characterization of the largest cohort of RT-associated bladder cancers to date. We show that bladder tumors developing after pelvic radiation are associated with distinct mutational signatures, which, along with *CDKN2A* loss and a history of smoking or a smoking-related mutational signature, have a poor prognosis. These data also suggest a need for a prospective study to determine if patients with high-risk clinical features (e.g., smoking and/or occupational exposures) may benefit from noninvasive genomic testing, prior to pelvic radiation, to guide prostate cancer treatment decisions.

## Materials and Methods

### Patient cohort

Patients with muscle-invasive bladder cancer and a prior history of RT for prostate cancer were retrospectively identified from two institutions [University of North Carolina (UNC) and Icahn School of Medicine at Mount Sinai] using pathology databases, electronic medical records, and clinical tumor board list review for inclusion into the RT-associated bladder cancer cohort. Included patients were diagnosed with bladder cancer between January 01, 2000, and May 15, 2015, and had tumor tissue available for next-generation sequencing (NGS). In addition, all patients at UNC with bladder cancer that had received standard-of-care NGS via the Foundation Medicine (FMI) FoundationOne CDx were reviewed and annotated for inclusion into the radiation-associated cohort or a CTRL cohort of non–RT-associated bladder cancer. Patients who were sequenced using deprecated bait sets or diagnosed with upper tract urothelial carcinoma were excluded from the analysis. Written informed consent was provided by all the study participants, and the protocol was approved by the participating institutional review boards: UNC Institutional Review Board and Icahn School of Medicine at Mount Sinai Institutional Review Board.

### Clinical annotation

Demographic and clinical information was annotated by each institution and de-identified prior to sending to the lead institution (UNC) for analysis. Clinical variables for the RT-associated cohort included the type of RT received (external beam, brachytherapy, or both) and time from RT to diagnosis of bladder cancer. Overall survival (OS) was calculated from the date of diagnosis of bladder cancer to date of death from any cause or last follow-up. Reported tumor–node–metastasis staging represents the staging at the time of initial diagnosis. Smoking status was recorded at the time of bladder cancer diagnosis.

### DNA sequencing

DNA was isolated from two to four formalin-fixed, paraffin-embedded slides per sample using the AllPrep DNA/RNA FFPE Kit (#80234 Qiagen), and quality and concentration were determined using TapeStation (Agilent). Sequencing was performed using hybrid capture–based NGS workflow for 324 cancer-related genes by FMI using FoundationOne CDx (Foundation Medicine, Inc.) in a Clinical Laboratory Improvement Amendments–certified, College of American Pathologists–accredited laboratory (Foundation Medicine; ref. 23). Copy number alterations along with the corresponding BAM files were then provided to UNC for downstream analysis (23, 24).

### Sequencing analysis

BAM files were converted to the FASTQ R1 and R2 files using the bamtofastq tool from biobambam2 v2.0.87 (25). The FASTQ files were aligned to the GRCh38 reference genome using BWA mem 0.7.17 (26), and then the BAM files were sorted, indexed, and duplicate reads were marked using the bamsormadup tool from biobambam 2.0.87. An in-house workflow was used to call somatic variants using Mutect2 (27) for single-nucleotide variants (SNV) and indels. Somatic variants were merged into a single variant call format (VCF) file and converted to a mutation annotation format (MAF) file using the tool vcf2maf v1.6.21 (28), and then the variants were annotated using a variant effect predictor (v103.1; ref. 29). Per sample somatic variants were merged and converted into a cohort-level somatic MAF file. Variants were then filtered down to those which passed the Mutect2 default filters, had a gnomADv3.1 allele frequency (AF) <5% (30), and were found within the ∼9,300 targeted genomic windows from the FoundationOne CDx capture. For recurrent mutation analysis, the MAF was further filtered for alterations with a variant allele frequency (VAF; t_alt reads/t_depth) of >5%.

Mutational signatures were derived based on all variants that passed the variant calling pipeline, inclusive of all classes and VAFs. Maftools was used to perform mutational signature analysis, and heatmaps were generated using pheatmap (31, 32).

### Validation datasets

Data from Mossanen and colleagues (17) were downloaded from https://github.com/CarvalhoFilipeL/CarvalhoFilipeL-Mouw_RABC. Mutational signatures and *KDM6A* calls were derived from the unfiltered MAF files. Copy number data, in the form of GISTIC2 values, were extracted from the supplemental data, and deletions were defined by a GISTIC2 score of less than −0.5. Outcome data were provided through request of the authors. The Cancer Genome Atlas (TCGA)–normalized gene expression and clinical variables were accessed through cBioPortal (https://www.cbioportal.org/study/summary?id=blca_tcga_pan_can_atlas_2018).

### Statistical analysis

Statistical analysis was performed using R version 4.2.2 and RStudio 2022.12.0 + 353, unless specifically noted. All packages used in the analysis were publicly availably either through https://www.r-project.org/ or www.bioconductor.org. Boxplots are represented by the IQR and midline at the median. Error bars equal the Q1/Q3 ± 1.5 × IQR. Categorical comparisons were made using either χ^2^ or Fisher exact test (as noted), dependent on the group size. Pairwise comparisons were performed using two-sided *t* test or Wilcoxon rank sum test (in cases of non-normal distribution). Multiple comparison correction was performed using Bonferroni correction. Kaplan–Meier plots were generated for survival analysis with significance determined using a log-rank or Cox proportional hazards model, as indicated.

### Data availability

Processed genomic data are available through request of the authors. Raw sequencing data were provided through a research agreement with FMI Inc. and are not available for public use through the authors.

## Results

### Patient cohort and clinical characteristics

**Figure 1:**
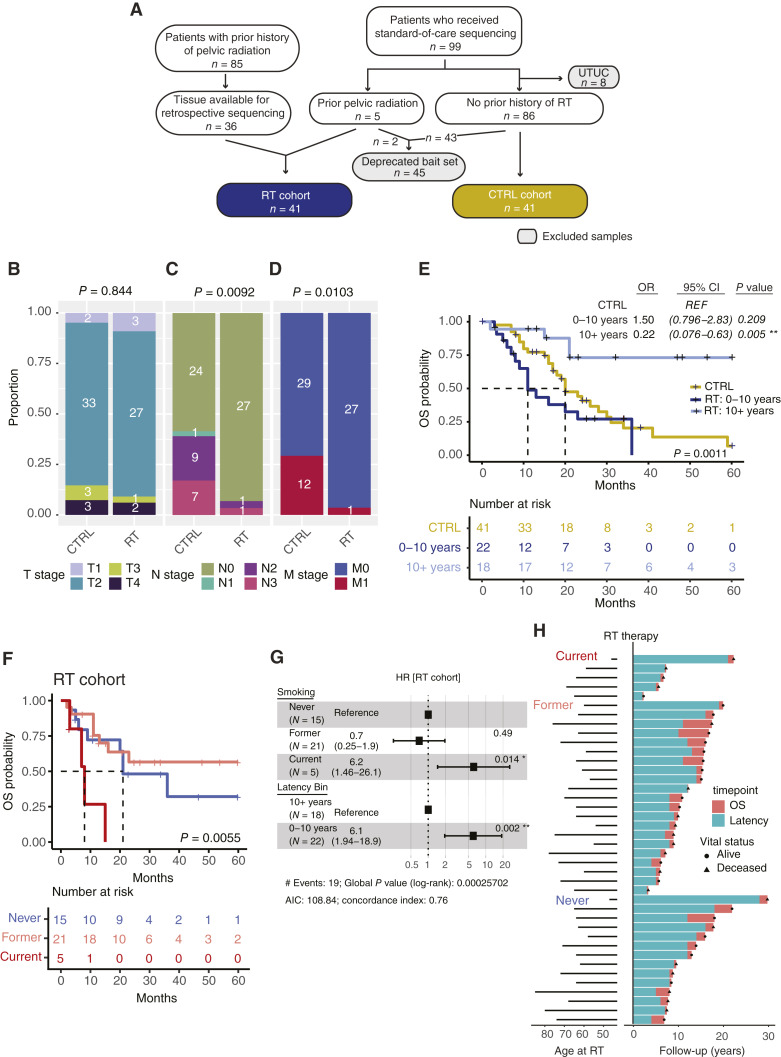
Construction and clinical characterization of the RT and CTRL cohorts. **A,** Flowchart representing identification and filtering of patients included or excluded in each cohort. Barplots of distribution of (**B**) T, (**C**) N, and (**D**) M stage tumors for both the CTRL and RT cohorts. Bar height represents the proportion of cohort with the absolute number inset in the respective box. Fisher exact test was performed to calculate significance between CTRL and RT sample distribution. **E,** Kaplan–Meier (KM) curves representing OS for patients with CTRL (gold), RT–short latency (dark blue), and RT–long latency (light blue) tumors. **F,** KM curves representing OS for RT patients grouped by smoking status at the time of the bladder cancer diagnosis. For KM curves, dashed lines indicate median survival. Significance was calculated using the Cox proportional hazard model with CTRL and never smokers as the reference, respectively. **G,** Forest plot visualizing a multivariate Cox proportional model for OS incorporating smoking and latency. Reference groups are indicated within the figure. **H,** Swimmer plot showing time from RT to end of follow-up. Solid black lines represent the age of patients at the time of prostate RT. The x-axis origin denotes the time of RT, with the teal bar length proportional to the latency and the pink bar indicating length of follow-up to death or last follow-up. Vital status is noted by either a circle (alive at last follow-up) or a triangle (deceased). UTUC; upper tract urothelial carcinoma.

From an initial pool of 85 patients who had previously received RT for prostate cancer, 35 patients had tissue available for DNA sequencing. An additional 99 patients who had undergone FMI sequencing as part of their care for bladder cancer within the UNC Health System were eligible for inclusion, with seven patients having had prior pelvic radiation and 92 patients having no prior history of pelvic radiation. As this was a retrospective study with a wide eligibility time range, the FoundationOne CDx assay evolved during the study window. To this end, we focused the analysis on the single bait set that allowed for the inclusion of the maximum number of patients across both the RT and CTRL cohorts. In total, the final cohort included 82 patients (RT = 41 and CTRL = 41; Fig. 1A; Supplementary Table S1).

Because the RT group was defined by prior pelvic RT for prostate cancer, all patients were male [*n* = 41 (100%)], whereas the CTRL cohort included male and female patients [male = 38 (68.3%)]. The median time from prostate treatment to bladder cancer diagnosis (latency) was 8.5 years (mean = 10.1, range = 2–28 years). Of the patients who received RT, 42% received brachytherapy, 31% received external beam, and 27% received combination. Race and smoking status were similar between the CTRL and RT groups. Within the RT group, latency was not associated with RT type, race, or smoking status (Supplementary Fig. S1A–S1C); however, there was a trend toward former and never smoking having a longer latency (median latency: current = 6 years, former = 9 years, never = 10.5 years).

Cohort-wide, patients with RT-associated bladder cancer were older than CTRL cohort (median age 75 vs 66 years, respectively; *P* value = 2e−4; Table 1). In the CTRL cohort, the median age of diagnosis for female patients was 5 years earlier than for males (female = 64 years, male = 69 years; *P* value = 0.1); however, after restricting the CTRL cohort to male patients, the RT cohort still had a significantly older age at diagnosis (CTRL = 69 years, RT = 75 years; *P* value = 0.01; Supplementary Fig. S1D–S1F).

**Table 1:** Summary of clinical variables for the control (CTRL) and radiation therapy (RT) cohorts.

Variable	CTRL (*N* = 41)^a^	RT (*N* = 41)^a^	*P* value^b^
Age at bladder cancer diagnosis (years)	69 (63, 75)	75 (72, 79)	**<0.001**
Sex			**<0.001**
Female	13 (32%)	0 (0%)	
Male	28 (68%)	41 (100%)	
Self-reported race			0.8
Asian	2 (5.0%)	0 (0%)	
Black	8 (20%)	8 (21%)	
Other	1 (2.5%)	1 (2.6%)	
White	29 (73%)	30 (77%)	
Unknown	1	2	
Smoking status (breast cancer Dx)			0.7
Current	4 (9.8%)	5 (12%)	
Former	25 (61%)	21 (51%)	
Never	12 (29%)	15 (37%)	
cTstage			0.8
T1	2 (4.9%)	3 (9.1%)	
T2	33 (80%)	27 (82%)	
T3	3 (7.3%)	1 (3.0%)	
T4	3 (7.3%)	2 (6.1%)	
Unknown	0	8	
cNstage			**0.009**
N0	24 (59%)	27 (93%)	
N1	1 (2.4%)	0 (0%)	
N2	9 (22%)	1 (3.4%)	
N3	7 (17%)	1 (3.4%)	
Unknown	0	12	
cMstage			**0.007**
M0	29 (71%)	27 (96%)	
M1	12 (29%)	1 (3.6%)	
Unknown	0	13	
Prostate cancer	4 (9.8%)	41 (100%)	**<0.001**
RT type			**<0.001**
Both	0 (0%)	11 (27%)	
Brachy	0 (0%)	17 (41%)	
External beam radiation therapy (EBRT)	0 (0%)	8 (20%)	
None	41 (100%)	0 (0%)	
Unknown	0 (0%)	5 (12%)	
Tumor latency			**<0.001**
0–10 years	0 (0%)	22 (55%)	
10+ years	0 (0%)	18 (45%)	
CTRL	41 (100%)	0 (0%)	
Unknown	0	1	

Significant *P* values are bolded

a Median (IQR); *n* (%).

b Wilcoxon rank sum test; Pearson χ^2^ test; Fisher exact test.

There was no association between smoking and age of diagnosis among males within the CTRL cohort (*P* value > 0.51), nor was smoking associated with the age of the patient at the time of RT for prostate cancer (*P* value > 0.2; Supplementary Fig. S1G and S1H, Table 2). However, among the RT cohort, current smokers were diagnosed with bladder cancer at a significantly younger age than former and never smokers (current = 67 years, former = 75 years, never = 76 years; *P* values vs. former and never smokers <0.01 and 0.001, respectively; Supplementary Fig. S1I).

**Table 2:** Summary of clinical variables within the radiation therapy cohort, grouped by latency.

Variable	0–10 years (*N* = 22)^a^	10+ years (*N* = 18)^a^	*P* value^b^
Age at bladder cancer diagnosis (years)	74 (69, 78)	76 (74, 80)	0.15
Tumor latency	6.5 (5.0, 8.0)	13.5 (12.0, 16.0)	**<0.001**
Self-reported race			0.7
Black	5 (24%)	3 (18%)	
Other	0 (0%)	1 (5.9%)	
White	16 (76%)	13 (76%)	
Unknown	1	1	
Smoking status (breast cancer Dx)			0.5
Current	4 (18%)	1 (5.6%)	
Former	11 (50%)	10 (56%)	
Never	7 (32%)	7 (39%)	
RT type			0.5
Both	4 (18%)	6 (33%)	
Brachy	10 (45%)	7 (39%)	
EBRT	4 (18%)	4 (22%)	
Unknown	4 (18%)	1 (5.6%)	
cTstage			0.6
T1	1 (5.3%)	2 (15%)	
T2	15 (79%)	11 (85%)	
T3	1 (5.3%)	0 (0%)	
T4	2 (11%)	0 (0%)	
Unknown	3	5	
cNstage			0.7
N0	15 (94%)	11 (92%)	
N2	1 (6.3%)	0 (0%)	
N3	0 (0%)	1 (8.3%)	
Unknown	6	6	
cMstage			>0.9
M0	15 (94%)	11 (100%)	
M1	1 (6.3%)	0 (0%)	
Unknown	6	7	

Significant *P* values are bolded

a Median (IQR); *n* (%).

b Wilcoxon rank sum test; Fisher exact test.

### Tumor latency and smoking are associated with worse OS in RT-associated bladder cancer

To evaluate if prior radiation conferred a worse prognosis among patients with bladder cancer, we compared the OS between the CTRL and RT cohorts. The median OS for the CTRL cohort was 20 months and for RT-associated bladder cancer was 23 months [log-rank *P* value = 0.27; HR 0.72; 95% confidence interval (CI), 0.41–1.3; Supplementary Fig. S2A]. Upon review of the tumor–node–metastasis staging, no difference was seen in T stage (range T1–T4; *P* = 0.08), whereas the CTRL cohort was enriched for patients with N and M stages >0 (*P* = 0.009 and *P* = 0.01, respectively; Fig. 1B–D). This would be expected as within the timeframe of case review, NGS sequencing was only performed as standard of care for those patients with advanced or metastatic disease.

Among RT-associated tumors, latency calculated from the time of pelvic RT to bladder cancer diagnosis was a significant prognostic feature. Patients with a longer latency (RT ≥ 10 years, *n* = 18) had a significantly better survival than both those with a shorter latency (RT < 10 years; *n* = 22; HR 6.8; 95% CI, 2.22–21.07; *P* = 0.001) and those with no prior radiation (HR 4.6; 95% CI, 1.58–13.11; *P* = 0.005; Fig. 1E). As the short latency group trended toward worse prognosis than the CTRL tumors, but did not reach significance, we leveraged TCGA for a further comparator group. Recapitulating the survival trends seen in the UNC cohort, long latency tumors trended toward better prognosis (HR 0.41; 95% CI, 0.15–1.1; *P* = 0.082), whereas the short latency tumors had a significantly worse prognosis than TCGA (HR 2.41; 95% CI, 1.42–4.1; *P* = 0.001; Supplementary Fig. S2B).

To further validate latency findings, we similarly split a RT bladder cohort from Dana–Farber Cancer Institute (DFCI), which only consisted of an RT arm, into short and long latency groups (17), and the short latency group had a median survival which was about 6 months less than that of the long latency group (767 vs. 975 days; Supplementary Fig. S2C), although this difference did not reach statistical significance.

Prior work in breast and lung cancer has demonstrated that smoking while undergoing RT is associated with worse outcomes and smoking while undergoing RT for breast cancer is associated with an increased risk of a secondary lung cancer (33–39). To evaluate if a similar phenomenon existed between pelvic radiation and bladder cancer, patients were divided into their respective CTRL and RT groups and stratified by smoking status (current, former, and never smokers). Within the CTRL cohort as well as TCGA, smoking was not associated with a decreased OS (Supplementary Fig. S2D–S2F). However, within the RT cohort, current smokers had a worse prognosis than both former (HR 0.152; 95% CI, 0.05–0.56; *P* = 0.004) or never smokers (HR 0.223; 95% CI, 0.06–0.816; *P* = 0.023; Fig. 1F). In a multivariate model of survival, smoking and latency were both independently prognostic (*P* = 0.014 and *P* = 0.002, respectively; Fig. 1G). Additionally, whereas smoking was not significantly associated with decreased latency, time from RT to last follow-up (latency + OS), current smokers had a numerically reduced RT to death interval as compared with former or never smokers (median survival = current – 6.96 years, former – not reached, and never – 17.75 years; Fig. 1H).

### Low VAF alteration and *KDM6A* splice mutations are enriched in the RT cohort

**Figure 2:**
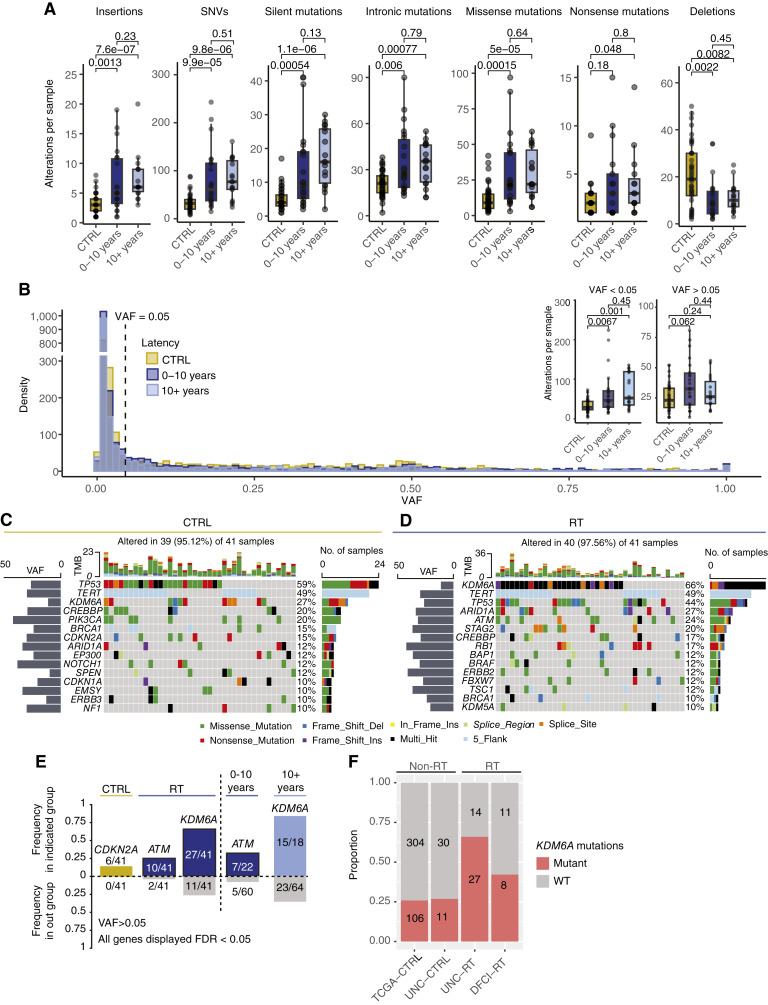
Mutational characterization of the CTRL and RT cohorts. BAM files corresponding to the FoundationOne sequencing assay were back converted to FASTQ format and aligned to GRCh38. **A,** The number of alterations per sample was calculated and plotted by variant type (insertions, SNVs, silent, intronic, missense, nonsense, and deletions). Pairwise comparisons between CTRL (gold), RT–short latency (dark blue), and RT–long latency (light blue) tumors were made using the Wilcoxon rank-sum test, with *P* values listed above their corresponding comparison. **B,** Histogram of VAF (alternate read count/total read depth) for CTRL (gold), RT–short latency (dark blue), and RT–long latency (light blue) tumors, variants are bin by a frequency of 0.01 (1%). Dashed line represents a VAF of 0.05 (5%). Inset boxplots represent alterations per sample either above or below the 5% VAF threshold and grouped by latency. Pairwise comparisons between CTRL (gold), RT–short latency (dark blue), and RT–long latency (light blue) tumors were made using the Wilcoxon rank-sum test, with *P* values listed above their corresponding comparison. Boxplots are represented by the IQR and midline at the median. Error bars equal the Q1/Q3 ± 1.5 × IQR. Oncoplots of recurrent mutations occurring above a frequency of 10% in the (**C**) CTRL and (**D**) RT cohorts. Only mutations with a VAF of ≥5% were included. **E,** Mutation enrichment was performed using the maftools package, and genes with an FDR >0.05 are indicated. Bars to the left of the dashed line represent CTRL vs. RT, with bars to the right of the dashed line indicating significant gene based on a 1 vs. other comparison. **F,** Comparison of *KDM6A* mutations between non-RT (TCGA and CTRL) and RT (RT and DFCI) cohorts. WT, wild type.

We next assessed RT-specific genomic alterations within our cohort. To understand the full distribution of variants, we first analyzed the number of alterations per sample by variant class (e.g., SNV and deletion) inclusive of any VAF. On a per sample basis, the RT cohort contained increased numbers of insertions and SNPs, but not deletions (Fig. 2A). As no VAF filter had been applied, we next wanted to evaluate if the increased number of alterations per sample within the RT cohort was restricted to low VAF samples. Indeed, RT samples had a significantly higher number of low VAF alterations per sample than the CTRL cohort (*P* = 0.007 and *P* = 0.001; 0–10 years and 10+ years vs. CTRL, respectively; Fig. 2B). To better understand the gene-level mutation specificity between our cohorts, we performed recurrent mutation analysis on genes with VAF >0.05 (restricting our analysis to genes more likely associated with clonal growth). Overall, mutation frequencies were consistent between the CTRL and RT cohorts, in line with prior muscle-invasive bladder cancer (MIBC) genomic results (40, 41). The most frequently mutated genes included (CTRL %; RT%) *TP53* (54%; 49%), *TERT* promoter mutations (49%; 49%), *CDKN2A* (12%; 27%), and *CREBBP* (27%; 20%; Fig. 2C and D). Of the 311 genes evaluated, only *CDKN2A* (CTRL), *ATM* (RT: 0–10 years), and *KDM6A* (RT: 10+ years) were significantly differentially mutated (*P* < 0.05; Fig. 2E; Supplementary Table S2). Of the three differentially mutated genes, only *KDM6A* displayed recurrently mutated regions associated with RT, located proximal to splice sites (Supplementary Fig. S3A–S3C). To validate that *KDM6A* alterations occurred at an increased frequency within RT-associated bladder cancer, we calculated the proportion of *KDM6A* altered tumors in the DFCI validation cohort and in a non-RT cohort, TCGA bladder study (17). The frequency of *KDM6A* alteration within TCGA was similar to that of our CTRL cohort (TCGA = 26%, CTRL = 27%), whereas the frequency within the DFCI RT cohort was 18% greater (44%; Fig. 2F). Taken together, this points to a broad subclonal pattern of DNA damage induced by RT to the normal urothelium, along with few distinct recurrent RT-induced driver mutations.

### RT-associated tumors have a unique mutational signature

**Figure 3:**
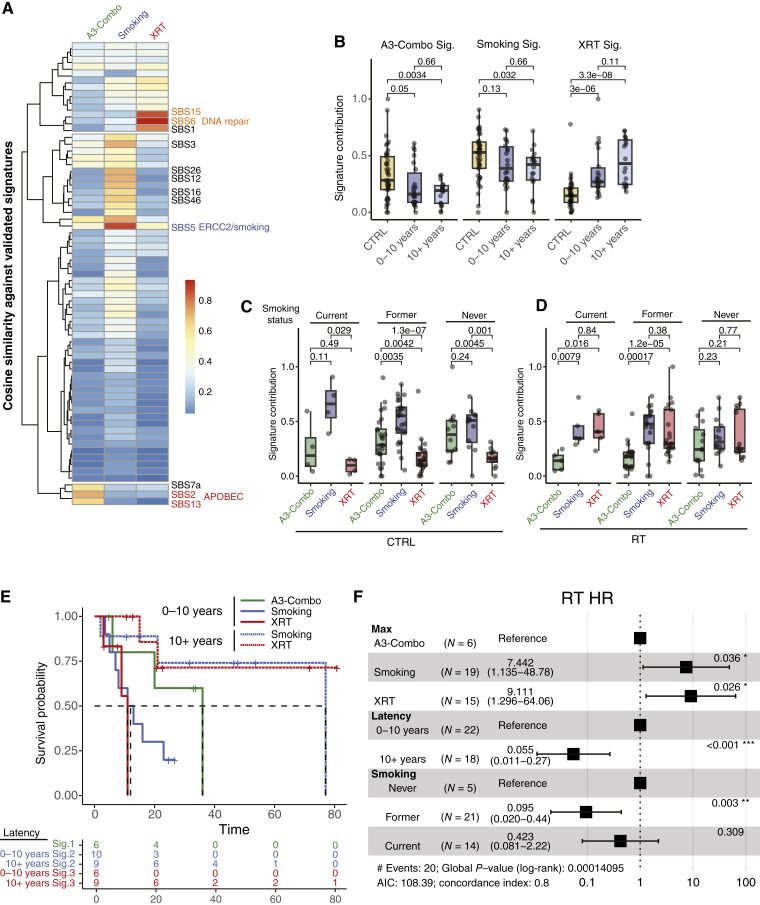
Identification of *de novo* mutational signatures. **A,** Heatmap of the cosine similarity between the *de novo* mutational signatures and known Catalogue of Somatic Mutations in Cancer SBS profiles. Boxplots of the signature contribution to the overall SNVs for each sample grouped by (**B**) signature and latency or (**C**) smoking status and signature for CTRL and (**D**) RT tumors. Pairwise comparisons between CTRL (gold), RT–short latency (dark blue), and RT–long latency (light blue) tumors were made using the Wilcoxon rank-sum test, with *P* values listed above their corresponding comparison. Boxplots are represented by the IQR and midline at the median. Error bars equal the Q1/Q3 ± 1.5 × IQR. **E,** Samples were grouped by the mutational signature with the greatest signature contribution. KM curves were generated based on this max signature assignment for OS for RT patients. Dashed lines indicate median survival. **G,** Forest plot visualizing a multivariate Cox proportional model for OS incorporating the max signature group, latency, and smoking status. WT, wild type.

Reproducible patterns of SNVs within a specific trinucleotide motif structure, referred to as mutational signatures, have been associated with various biological functions and environmental exposures (42). These SNVs within their flanking nucleotide context can be quantified and clustered to reveal recurrent mutational signatures specific to a cohort. Those cohort-specific signatures can then be correlated to a set of previously described signatures with known etiology to infer possible mechanism or biological significance. To determine if unique mutational signatures were present within the CTRL and RT cohorts, we quantified and clustered the mutational motifs and correlated the corresponding signatures back to those described in the Catalogue of Somatic Mutations in Cancer signature database. We identified three cohort-derived signatures: one most similar to the APOBEC activity signatures, SBS2 and SBS13 (A3-Combo); a second signature (smoking) with shared similarity to the *ERCC2* mutant/smoking signature, SBS5 (43, 44); and a third [radiation therapy signature (XRT)], which was only enriched within the XRT tumors and is most similar to defects in DNA mismatch repair, SBS6 and SBS15 (Fig. 3A; Supplementary Fig. S4A; Supplementary Table S3).

Following *de novo* signature identification, the number of mutations attributed to each signature was calculated (“signature contribution”). Whereas the smoking signature was consistent and accounted for the largest proportion of variants across the cohort, the A3-Combo signature was significantly increased within the CTRL cohort compared with RT (short and long latency; *P* = 0.05 and *P* = 0.003), and the XRT signature displayed a significant and stepwise increase from the CTRL to RT cohort (*P* = 3e−6 and *P* = 3e−8; Fig. 3B). In keeping with the etiologies of the correlated signatures, the smoking signature was significantly increased over that of the A3-Combo within current and former smokers while showing no differential contribution in the never smokers (Fig. 3C). Likewise, within the RT cohort, the XRT signature was present at equal levels within the current and former smokers, whereas all three signatures were equally represented within never smokers (Fig. 3D).

Finally, as KDM6A was the most frequently altered gene in the RT cohort, we extracted the trinucleotide motifs for each alteration, in both the CTRL and RT cohorts. When visualized by cohort, the RT samples had a noticeable increase in the frequency of A[C>T]N and C[C>T]N motifs, similar to that of the XRT signatures, in addition to an increased frequency of N[T>C]N motifs (Supplementary Fig. S4B). Taken together, these data are suggestive of the fact that RT confers a unique mutational signature and may account for the increased number of KDM6A mutations present in the RT samples.

### XRT and smoking signatures identify a subset of poor prognosis patients

Because current smokers and patients with short latency tumors had worse OS (Fig. 1F), we postulated that patients in which the smoking or XRT signature contributed to the majority of the tumor’s mutations would, in turn, have the worst prognosis. When patient survival was analyzed within the RT group by the max signature contribution, short latency tumors with smoking or XRT signature had the worst outcome, with the A3-Combo signature and long latency tumors having the best outcome (Fig. 3E). The smoking and XRT signatures retained their prognostic ability, even when correcting for smoking status and latency (smoking vs. A3: HR = 7.4; 95% CI, 1.14–48.78; *P* = 0.036; XRT vs. A3: HR = 9.1; 95% CI, 1.3–64.06; *P* = 0.026; Fig. 3F). Whereas there was no signature-specific survival within the CTRL cohort, this could be confounded by the fact that the patients were biased toward advanced disease (Supplementary Fig. S5A).

To validate the signature-specific survival within the RT samples, we repeated the mutational signature analysis in the independent DFCI cohort (17). Again, we identified three mutational signatures that corresponded to SBS2/SBS13, SBS5, and SBS6/SBS15, respectively (Supplementary Fig. S5B). Even though the DFCI signatures were highly similar to the same Catalogue of Somatic Mutations in Cancer signatures as UNC, we directly compared the motif patterns of the DFCI signatures and the UNC signatures. UNC A3 signature had the highest correlation to DFCI-A, the UNC smoking signature was most correlated to DFCI-B, and the UNC XRT signature was most correlated to DFCI-C while also showing similarity to DFCI-B (Supplementary Fig. S5C). The signature related to prognosis mirrored that of the UNC cohort: A good prognosis was associated with the A3-Combo signature, whereas the smoking signature (HR = 6.6; 95% CI, 1.14–39; *P* = 0.035) and XRT signature conferred a poor prognosis (HR = 3.5; 95% CI, 0.71–17; *P* = 0.123; Supplementary Fig. S5D).

### Chr9p21.3 loss is prognostic and further reduces survival in the context of RT

For more than 30 years, the field has appreciated that recurrent copy number events occur at a high frequency and are an early driver of bladder cancer (40, 45, 46). Chr9p21.3 loss (containing *CDKN2A*, *CDKN2B*, and *MTAP*) is the most frequent alteration, occurring in ∼53% of NMIBC tumors and 22% of MIBC (40, 47). To understand what, if any, focal amplifications or deletions are specific to RT-associated bladder tumors, we compared the FMI copy number calls between the CTRL cohort and the RT cohort. Of the 26 copy number events detected, only *PPARG* amplification was significantly enriched in the RT cohort (RT: *n* = 7, CTRL: *n* = 0; Fisher exact *P* = 0.01).

**Figure 4:**
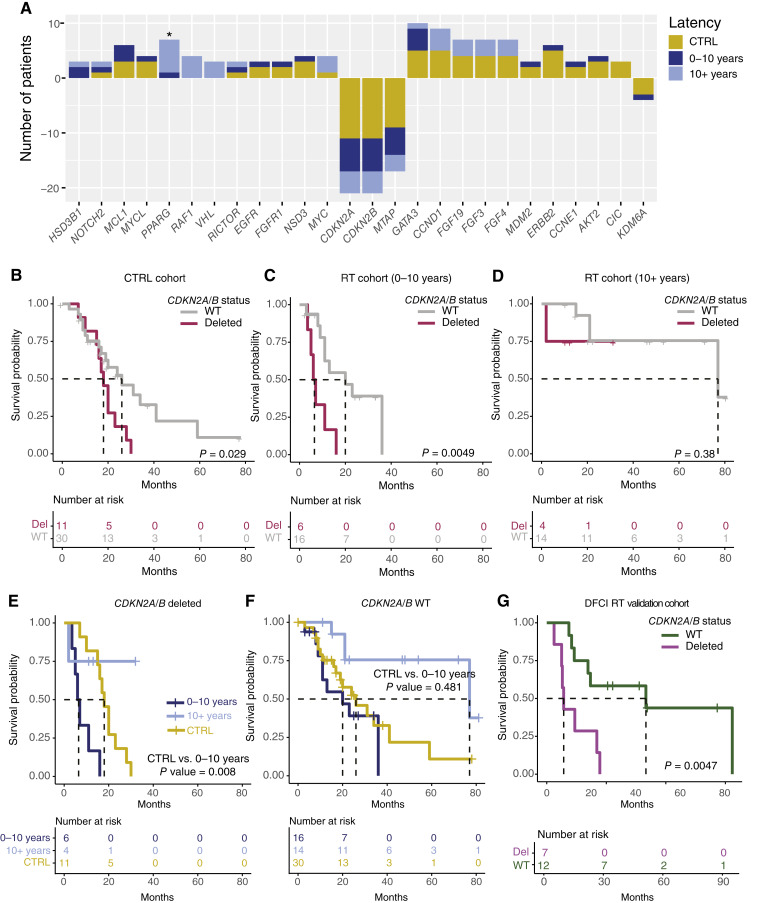
Comparison of the copy number landscape between CTRL and RT tumors. **A,** Copy number status, as determined by the FoundationOne CDx assay, was plotted by the number of tumors with a given alteration (gains = positive value, losses = negative values). Groups are shown as CTRL (gold), RT–short latency (dark blue), and RT–long latency (light blue). Significant enrichment was calculated using the Fisher exact test, and *P* < 0.05 is indicated by an asterisk (*). Patients were separated by latency, (**B**) CTRL, (**C**) short Latency, and (**D**) long latency, and OS differences between chr9p21.3 WT and loss (as determined by *CDKN2A/B* copy number status) were visualized using KM curves. Log rank *P* value was calculated for the pairwise comparison. Patients were then separated by *CKDN2A/B* copy number status to compare the difference in OS between the latency group within (**E**) deleted or (**F**) copy number neutral tumors. **G,** RT samples in the DFCI cohort were grouped by *CDKN2A/B* loss (GISTIC2 value <0.5), and OS was visualized. For all KM plots, dashed lines indicate median survival. WT, wild type.

*CDKN2A/CDKN2B* was the most frequent copy number event, occurring in 27% of CTRL samples (11/41) and 24% of RT samples (10/41; Fig. 4A; Supplementary Table S4). *CDKN2A/CDKN2B*-deleted tumors had decreased survival compared with copy number neutral tumors in both the CTRL (HR = 2.41; 95% CI, 1.07–2.43; *P* = 0.033) and short latency RT cohorts (HR = 4.58; 95% CI, 1.44–14.5; *P* = 0.01; Fig. 4B–D). Among the *CDKN2A/B*-deleted tumors, short latency RT tumors had a significantly worse OS compared with CTRL tumors (HR = 7.11; 95% CI, 1.89–26.7; *P* = 0.004); however, no difference was observed among CDKN2A/B copy number neutral tumors (Fig. 4E and F). Consistent with these findings, *CDKN2A/CDKN2B*-deleted tumors within the DFCI cohort displayed a 41-month reduced survival compared with wild-type tumors (HR = 4.65; 95% CI, 1.45–14.9; *P* = 0.01).

### Mutational signatures in conjunction with *CDKN2A* loss are prognostic, independent of prior RT

To understand if the smoking and RT signatures are prognostic in a more general setting, we correlated the trinucleotide motif frequencies from TCGA bladder cancer study (BLCA) to our cohort-derived A3-Combo, smoking, and XRT signatures. Using the Pearson correlation coefficient as the similarity metric, we first validated that the smoking signature was associated with smoking status. Current smokers had a significantly higher correlation with the UNC smoking signature than former or never smokers (*P* = 0.013, *P* = 0.011, respectively), whereas smoking status was not associated with the A3 or XRT signatures (Supplementary Fig. S6A–S6C).

**Figure 5:**
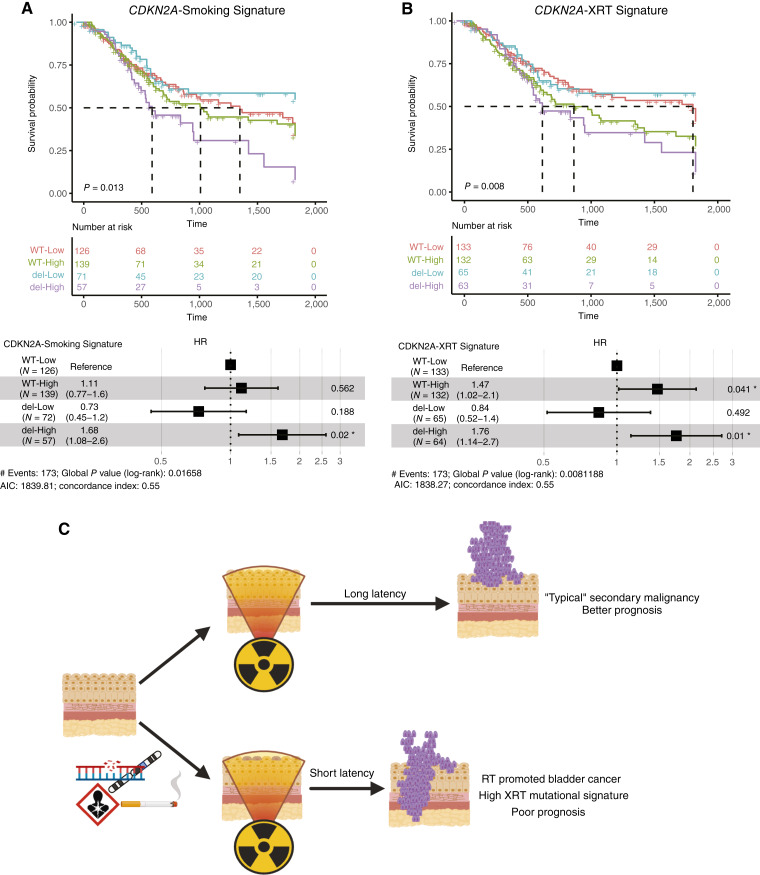
Prognostic value of the smoking and XRT signatures in TCGA and proposed scheme of RT-associated bladder cancer development. The Pearson correlation between the UNC signature motif frequency profile and TCGA BLCA samples was calculated, and TCGA samples were split into high and low groups at the median correlation. Samples were further split by *CDKN2A* status, and KM curves were generated. A multivariate Cox proportional hazard model was calculated for both the (**A**) smoking signature and (**B**) XRT signature. For all KM plots, dashed lines indicate median survival. **C,** Proposed model for the interplay between preexisting alteration in the urothelium and RT. WT, wild type.

Next, TCGA tumors were split based on median correlation into high and low groups for each signature. The high correlation group for both the smoking (HR = 1.4; 95% CI, 1.05–1.9; *P* = 0.02) and XRT signatures (HR = 1.65; 95% CI, 1.22–2.23; *P* = 0.001) was associated with poor prognosis. Furthermore, specific to the XRT signature, when *CDKN2A* loss was integrated into the analysis, it further defined a subset of patients with worse prognosis (Fig. 5A and B; Supplementary Fig. S6D).

## Conclusion

This study demonstrates that prior RT is associated with increased numbers of low-frequency SNVs, which results in a RT-specific mutational signature. This signature, in addition to a smoking signature, *CDKN2A/B* loss, patient smoking status, and a tumor latency of <10 years represent a key set of molecular and clinical variables that characterize a tumor with poor prognosis following RT. Additionally, the smoking and XRT signatures may be able to identify tumors with a pattern of genomic alterations conferring a more aggressive phenotype, independent of the canonical recurrent mutations.

## Discussion

Previous studies have demonstrated that RT for the treatment of prostate cancer results in up to a 3-fold increased risk for the subsequent development of bladder cancer. Although the relative risk of developing a secondary bladder cancer is low, thousands of men are possibly at risk over the course of their lifetime, given that prostate cancer cases are projected to reach almost 300,000 new cases in 2024. The relatively low frequency of secondary bladder cancer has also made it difficult to construct cohorts of RT-associated bladder tumors to interrogate how the biology and genomics may differ from that of non-RT–associated bladder tumors. Here, we present the genomic analysis of, to our knowledge, the largest cohort of bladder tumors associated with pelvic radiation.

Through integration of clinical and genomic data, we identified RT-specific genomic features. Overall, RT tumors had increased levels of insertions and SNVs, with decreased levels of deletions. At first glance, these results seem to be at odds with prior work by Behjati and colleagues, which demonstrated an increased number of deletions in secondary malignancies (22). However, their cohort was constructed from, primarily, a mixed set of sarcomas (*n* = 9) and breast tumors (*n* = 3). Due to differences in the cohort, their data cannot be easily projected on to our current study. The Behjati data also show a heterogeneous composition of structural variants, with an increased number of deletions in RT-associated tumors; however, breast tumors, which have molecular similarities to bladder cancer (48, 49), showed a lower rate of deletions than sarcomas. Additionally, their data demonstrating increased deletions in radiated prostate tumors are from primary prostate tumors, not the subsequent bladder malignancy, and thus not comparable with our cohort.

Interestingly, we identified an increased number of *KDM6A* mutations located within the splice regions. This alteration has not, to our knowledge, been previously described in the literature, which may be in part because of the lack of radiation-associated bladder cancer cohorts. Additionally, within the RT cohort, the *KDM6A* variants occurred as part of motifs that were enriched within the XRT mutational signature. This suggests that RT may, in part, be accounting for the increased number of *KDM6A* alterations, although future experimental validation will be required to establish a direct relationship and potential mechanism.

Minimal copy number differences were observed between the CTRL and RT samples; however, amplification of *PPARG* was significantly enriched within the RT samples. Previously, *PPARG* structural variants have been reported in thyroid cancer stemming from environmental radiation exposure (50, 51). In a bladder-specific context, *PPARG* expression is associated with urothelial differentiation and the better outcome LumP molecular subtype; therefore, it was of note that its amplification was specific to the long latency, better prognostic subset of tumors (40, 48, 49, 52, 53). Further exploration of these genetic alterations and their relationship to radiation is merited, as they could be predictive and prognostic for RT-associated bladder cancer.

Through global analysis of SNVs, we identified three mutational signatures associated with the CTRL and RT cohorts. These signatures, in addition to the loss of *CDKN2A*, demonstrated prognostic value in our discovery dataset along with two independent datasets. Earlier work by the Verhaak group demonstrated that RT-related genomic alterations, including *CKDN2A* loss, conferred poor prognosis in patients with glioblastoma who developed recurrence postradiation (54). Additionally, previous studies have suggested that *CDKN2A* loss could be predictive of immunotherapy response and progression, as well as prognostic value in bladder cancer (55–58). Similarly, we see poor prognosis among patients whose tumors have increased levels of the XRT-related signature and/or *CDKN2A* loss, if combined with prior pelvic radiation. When we applied the mutational signatures to a non–RT-specific cohort, TCGA, the signatures were again both prognostic, XRT alone and smoking in combination with *CDKN2A* loss. These data indicate that in the absence of RT or known smoking history, tumors that share a similar mutational pattern to that of patients treated with RT and/or smoking may have more aggressive disease. This suggests that even without patient-reported exposure histories, the molecular profile of the tumor may be informative, and further work is needed to understand the etiology of these exposure-related signatures.

When combining these results with the described relationship among smoking status, RT, and survival, we postulate that there are two distinct pathways that lead to the development of bladder cancer following pelvic radiation for prostate cancer. The first occurs in patients who have a baseline clinical history conferring a high risk for bladder cancer (e.g., heavy smoking/occupational exposure to known carcinogens) and receive RT for prostate cancer. The urothelium in these patients, prior to radiation, may have already undergone field cancerization (i.e., phenotypically normal cells that are primed for cancer formation due to prior mutagenic insults; ref. 59). In this setting, the off-target DNA damage induced by the radiation acts to hasten the development of the bladder cancer in the “pre-cancerized” bladder urothelium, ultimately leading to a short latency and poor prognosis. In the second pathway, patients who receive RT and later develop bladder cancer, in the absence of known bladder cancer risk factors, and have minimal preexisting urothelial genomic alteration are more likely to have a longer latency (10+ time course) tumor and ultimately a better prognosis.

There were several limitations to our study, which will need to be addressed in future work. One limitation, which points to a larger issue within the field, is the lack of tumor specimens available. Our study, which was the largest to date, included 41 individuals, which more than doubled the number of samples from the most recent prior study (*n* = 19; ref. 17). With evidence that RT may lead to more aggressive tumors in a subset of patients, institutions must collaborate to compile harmonized and clinically annotated cohorts to allow for a comprehensive evaluation of radiation-associated bladder cancer.

Although we observed a significant reduction in OS among current smokers within the RT cohort, the impact of the finding is limited because of the small sample size (*n* = 5). This lack of power underscores the previously stated wider issue of low sample number and diversity, which is part and parcel to the study of rarer tumor types. This lack of diversity spans a wide array of clinical variables including social factors, such as smoking and urban/rural geography, as well as ancestry and ethnicity.

As this was a retrospective study, we leveraged preexisting clinical sequencing data that had been generated by FMI. The use of a preset gene panel limited our ability to detect somatic alteration that is of interest in the research setting but not yet utilized clinically (e.g., *ERCC2*). Additionally, this reduced the resolution of *de novo* mutational signature identification compared with using data derived from whole-exome or whole-genome sequencing. Whereas using a clinical sequencing assay limited the number of variants we were able to detect, it did allow for the use of archival specimens and previously sequenced individuals. The use of FMI also was a strength, as in most clinical settings, patients will undergo commercial targeted sequencing and not have whole-exome or whole-genome sequencing performed on their tumor, making our findings more translatable to the clinic. Even with the described limitations, we were able to validate our findings in external datasets and demonstrate the presence of prognostic features, which could be applied to RT and non-RT bladder tumors.

We hypothesize that patients with a clinical history conferring a high risk for bladder cancer may also be at an increased risk for RT-associated poor prognosis bladder tumors because of a precancerization phenotype within the urothelium. Future prospective studies will need to be conducted to fully understand the extent of DNA damage in the urothelium before and immediately following RT and how smoking may compound this damage.

In summary, we present the largest genomic cohort of RT-associated bladder cancer to date and provide evidence for worse bladder-specific outcomes among patients who are smokers, have a short latency tumor, or have evidence of specific mutational signatures. Additionally, our data suggest that clinicians and patients should consider smoking and environmental exposure history as a factor when deciding on the appropriate treatment protocol for prostate cancer.

## Supplementary Material

Supplementary Figure S1 Supplementary Figure S1

Supplementary Figure S2 Supplementary Figure S2

Supplementary Figure S3 Supplementary Figure S3

Supplementary Figure S4 Supplementary Figure S4

Supplementary Figure S5 Supplementary Figure S5

Supplementary Figure S6 Supplementary Figure S6

Supplementary Table S1 Supplementary Table S1

Supplementary Table S2 Supplementary Table S2

Supplementary Table S3 Supplementary Table S3

Supplementary Table S4 Supplementary Table S4
