# Supplementary material for: Poor Prognosis among Radiation-Associated Bladder Cancer Is Defined by Clinicogenomic Features
Source: Cancer Res Commun. 2024 Sep 4;4(9):2320–34. doi: 10.1158/2767-9764.CRC-24-0352 (PMC11372343; doi:10.1158/2767-9764.CRC-24-0352)
Supplement: Supplementary Figure S1 [file crc-24-0352_supplementary_figure_s1_supps1.pdf]

Supplementary Figure S1

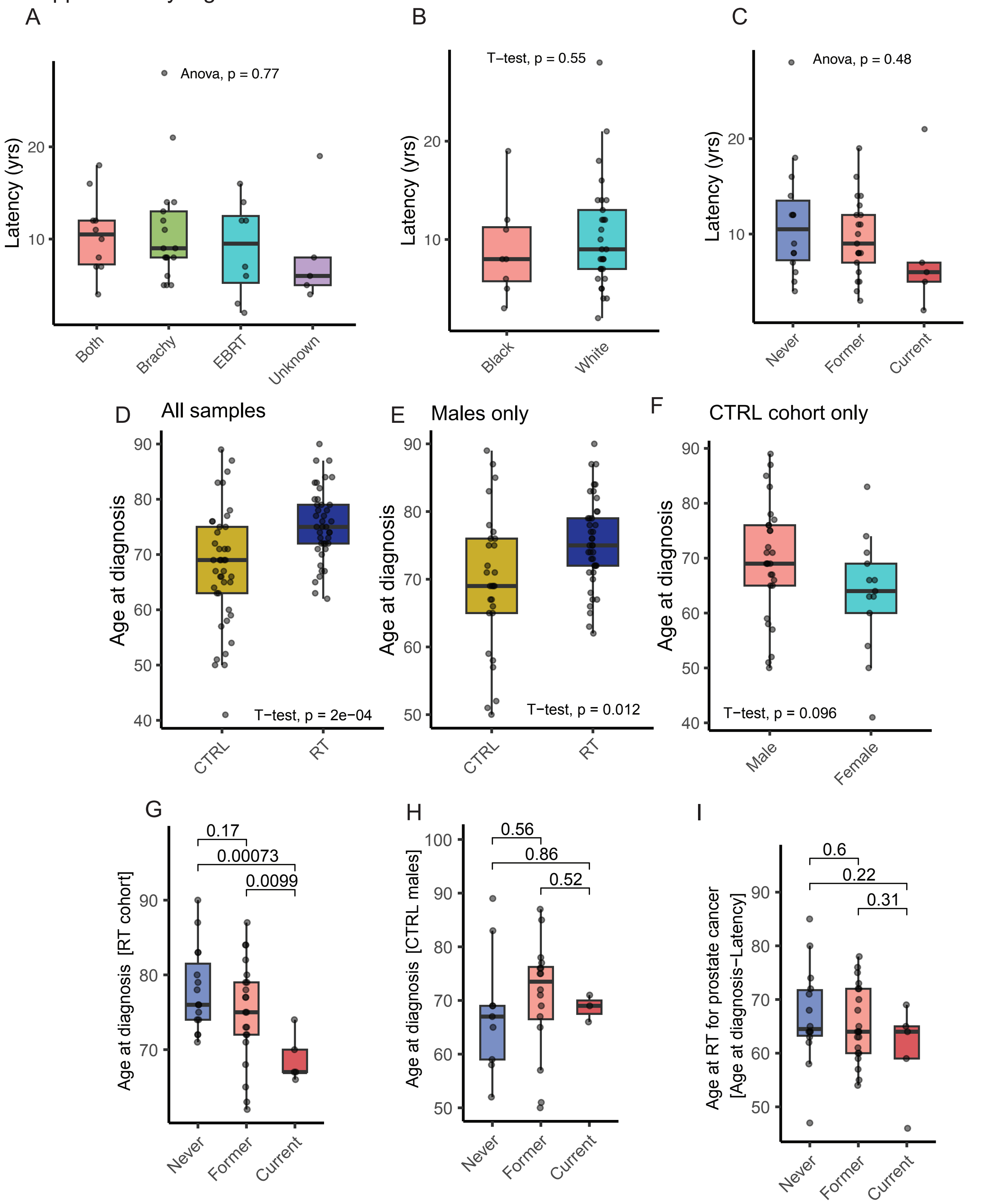

Supplementary Figure S1. Association between latency and age with clinical co-variables. Boxplots of latency by **(A)** type of radiotherapy, **(B)** race, and **(C)** smoking status. Boxplots of age at diagnosis grouped by **(D)** cohort and restricted to **(E)** males only. **(F)** Age of diagnosis was then split by sex for within CTRL comparison. Samples were group by smoking status and age of diagnosis for **(G)** RT and **(H)** CTRL were plotted along with **(I)** age at radiotherapy. Boxplots are represented by the IQR and midline at the median. Error bars equal the Q1/Q3 ± 1.5 × IQR.
