# Supplementary material for: Poor Prognosis among Radiation-Associated Bladder Cancer Is Defined by Clinicogenomic Features
Source: Cancer Res Commun. 2024 Sep 4;4(9):2320–34. doi: 10.1158/2767-9764.CRC-24-0352 (PMC11372343; doi:10.1158/2767-9764.CRC-24-0352)
Supplement: Supplementary Figure S2 [file crc-24-0352_supplementary_figure_s2_supps2.pdf]

Supplementary Figure S2

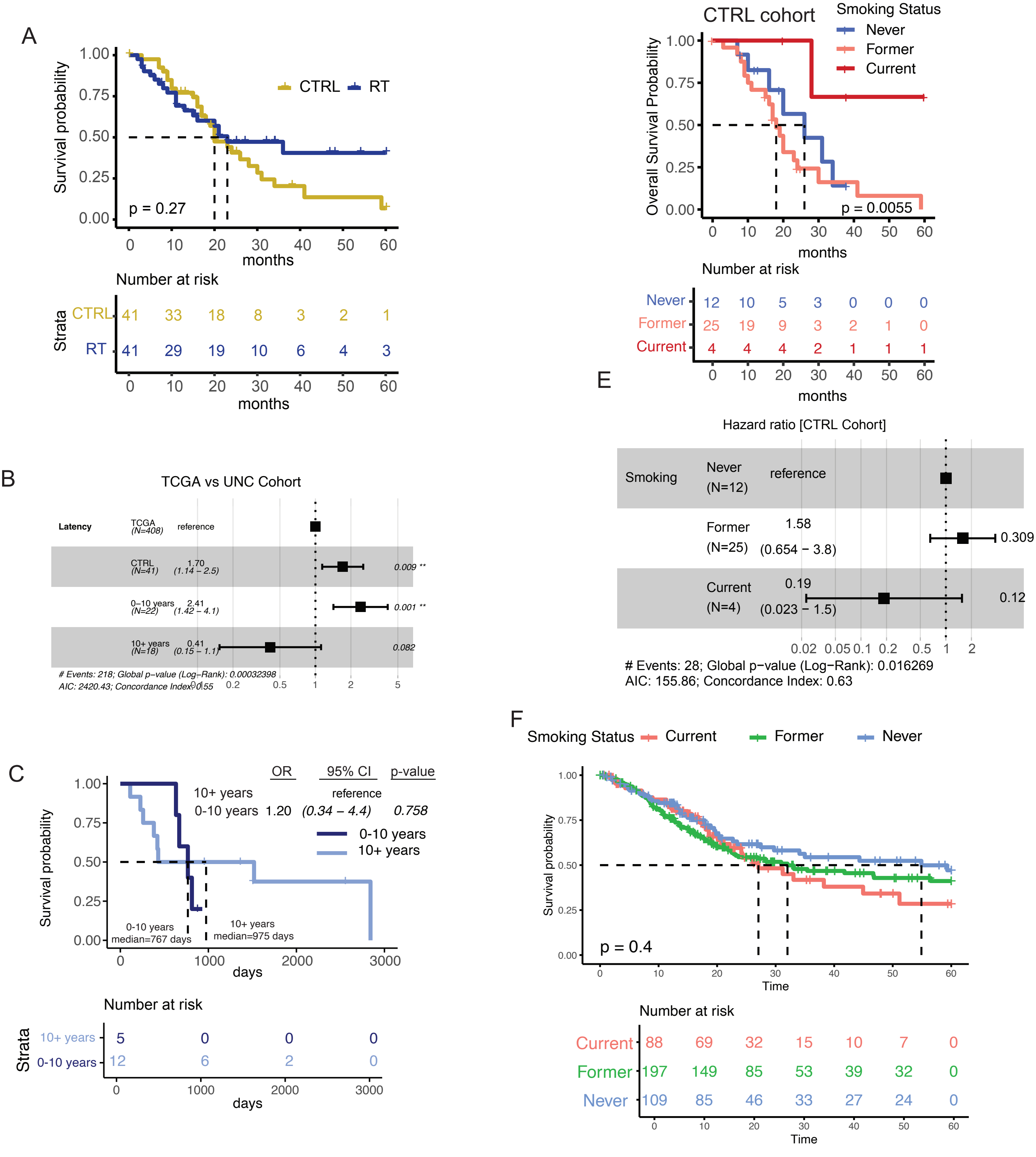

Supplementary Figure S2. Overall survival stratified by treatment and smoking status. (A) Kaplan-Meier (KM) curves representing overall survival for CTRL (gold), and RT (blue) cohorts. **(B)** Forest plot of TCGA and the UNC cohort divided by tumor latency. **(C)** Long and short latency KM curves representing overall survival from the DFCI validation cohort. **(D)** KM curves representing overall survival for CTRL patients with grouped by smoking status at the time of the bladder cancer diagnosis. For KM curves, dashed lines indicate median survival. **(E)** Forest plot visualizing the cox proportional model for overall survival grouped by smoking status. **(F)** TCGA cohort stratifed by smoking status.
