# Supplementary material for: Poor Prognosis among Radiation-Associated Bladder Cancer Is Defined by Clinicogenomic Features
Source: Cancer Res Commun. 2024 Sep 4;4(9):2320–34. doi: 10.1158/2767-9764.CRC-24-0352 (PMC11372343; doi:10.1158/2767-9764.CRC-24-0352)

Supplementary Figure S3

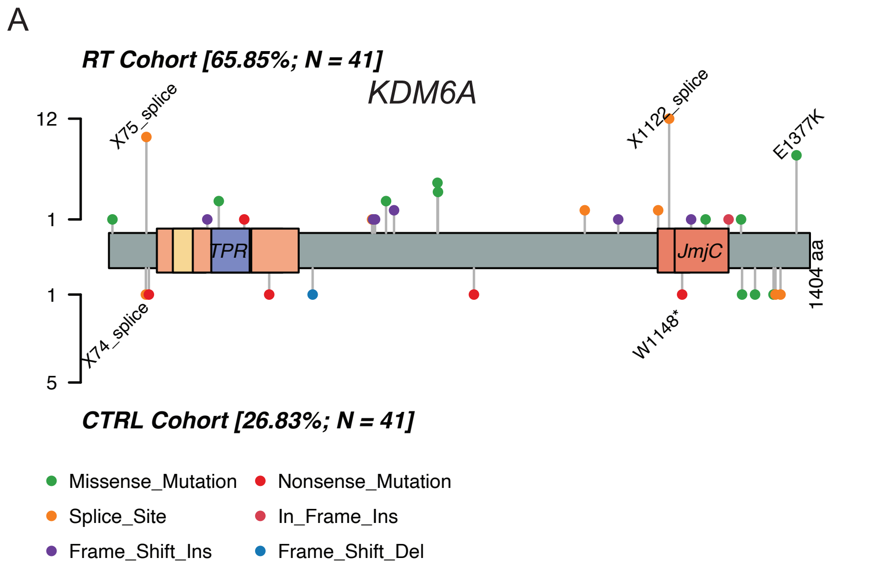

Supplementary Figure S3. Visualization of mutation sites for recurrently altered genes. The MafTools R package was used to generate lollipop plots to visualize recurrent point mutations and their distribution within the gene body for **(A)** KDMA6A, **(B)** ATM, **(C)** CDKN2A.

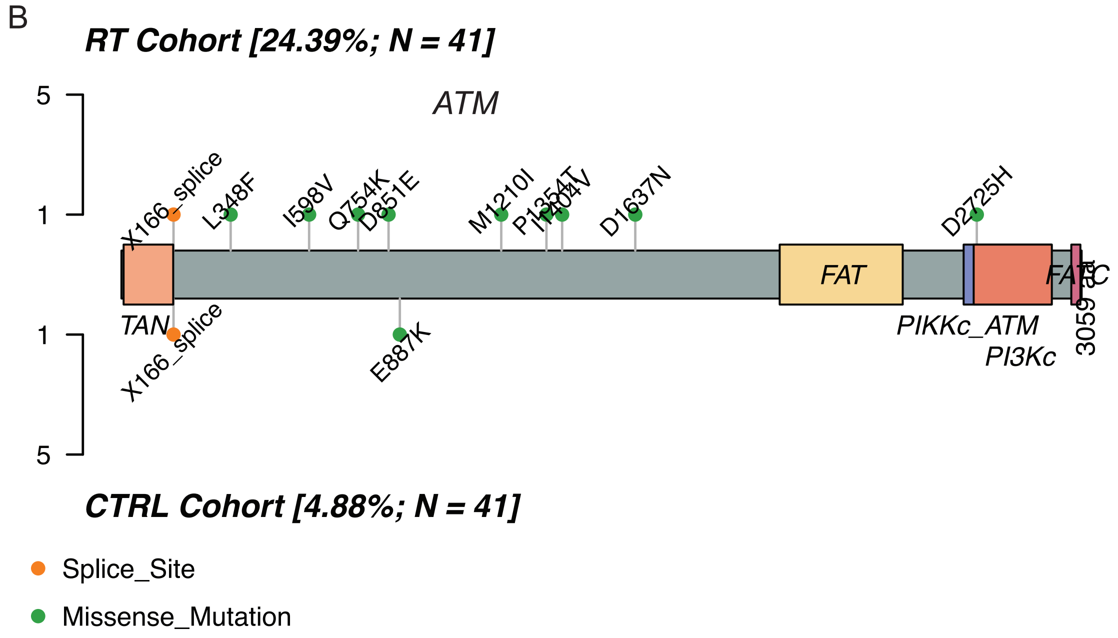

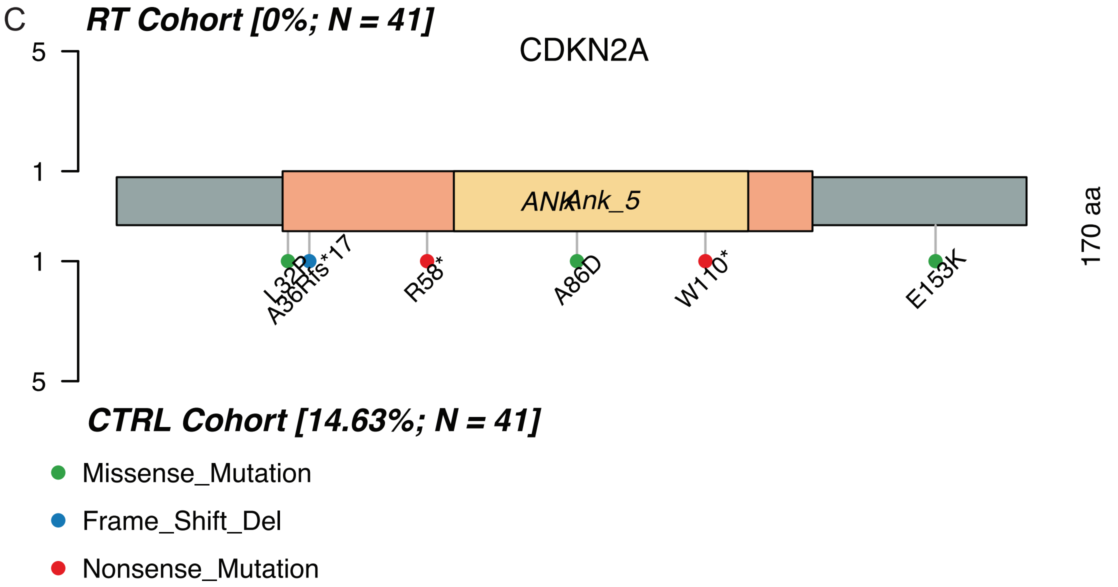

Supplement: Supplementary Figure S3 [file crc-24-0352_supplementary_figure_s3_supps3.pdf]
