# Supplementary material for: Poor Prognosis among Radiation-Associated Bladder Cancer Is Defined by Clinicogenomic Features
Source: Cancer Res Commun. 2024 Sep 4;4(9):2320–34. doi: 10.1158/2767-9764.CRC-24-0352 (PMC11372343; doi:10.1158/2767-9764.CRC-24-0352)
Supplement: Supplementary Figure S4 [file crc-24-0352_supplementary_figure_s4_supps4.pdf]

Supplementary Figure S4

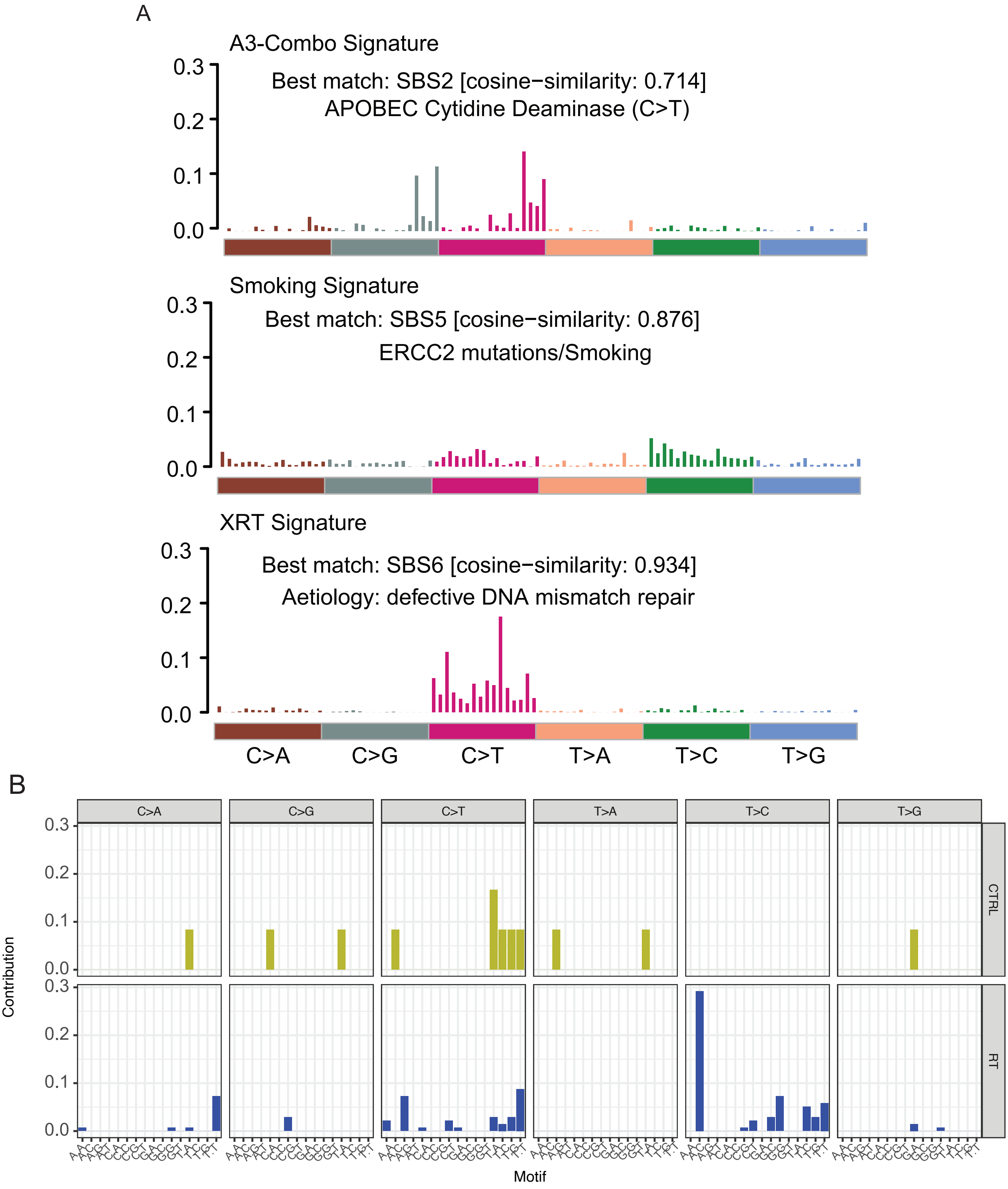

Supplementary Figure S4. Derivation and validation of mutational signatures. (A) Barplots representing the trinucleotide motif patterns that composed the A3, Smoking, and XRT signatures. Above each plot is the most similar COSMIC SBS signature with the corresponding cosine similarity. **(B)** Barplots representing the the trinueotide motif patterns present at each KDM6A alteration site. Samples are grouped by cohort.
