# Supplementary material for: Poor Prognosis among Radiation-Associated Bladder Cancer Is Defined by Clinicogenomic Features
Source: Cancer Res Commun. 2024 Sep 4;4(9):2320–34. doi: 10.1158/2767-9764.CRC-24-0352 (PMC11372343; doi:10.1158/2767-9764.CRC-24-0352)
Supplement: Supplementary Figure S5 [file crc-24-0352_supplementary_figure_s5_supps5.pdf]

Supplementary Figure S5

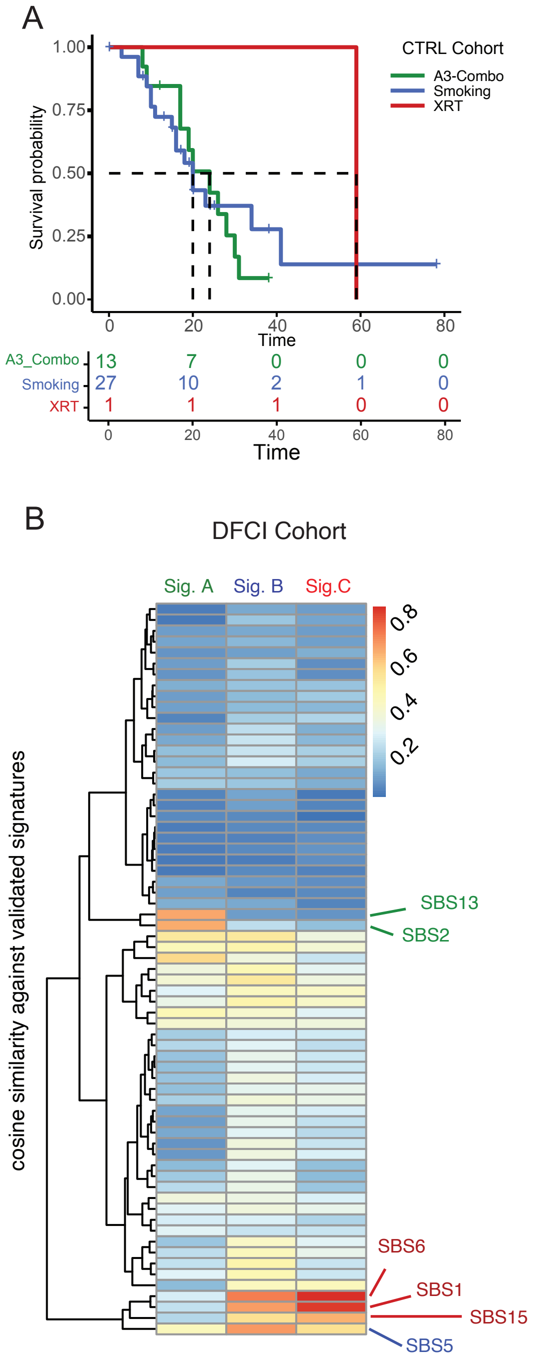

Supplementary Figure S5. Validation of mutational signatures. (A) KM plot of overall survival for the CTRL cohort grouped by their most prevalent mutational signature. **(B)** Heatmap of the cosine similarity for independently discovered mutational signatures within the DFCI validation cohort. **(C)** Heatmap of the Pearson correlation coefficient between the UNC and DFCI signature motif profiles. **(D)** KM plot of overall survival for the DFCI samples grouped by their most prevalent mutational signature.
