# Supplementary material for: Poor Prognosis among Radiation-Associated Bladder Cancer Is Defined by Clinicogenomic Features
Source: Cancer Res Commun. 2024 Sep 4;4(9):2320–34. doi: 10.1158/2767-9764.CRC-24-0352 (PMC11372343; doi:10.1158/2767-9764.CRC-24-0352)
Supplement: Supplementary Figure S6 [file crc-24-0352_supplementary_figure_s6_supps6.pdf]

Supplementary Figure S6

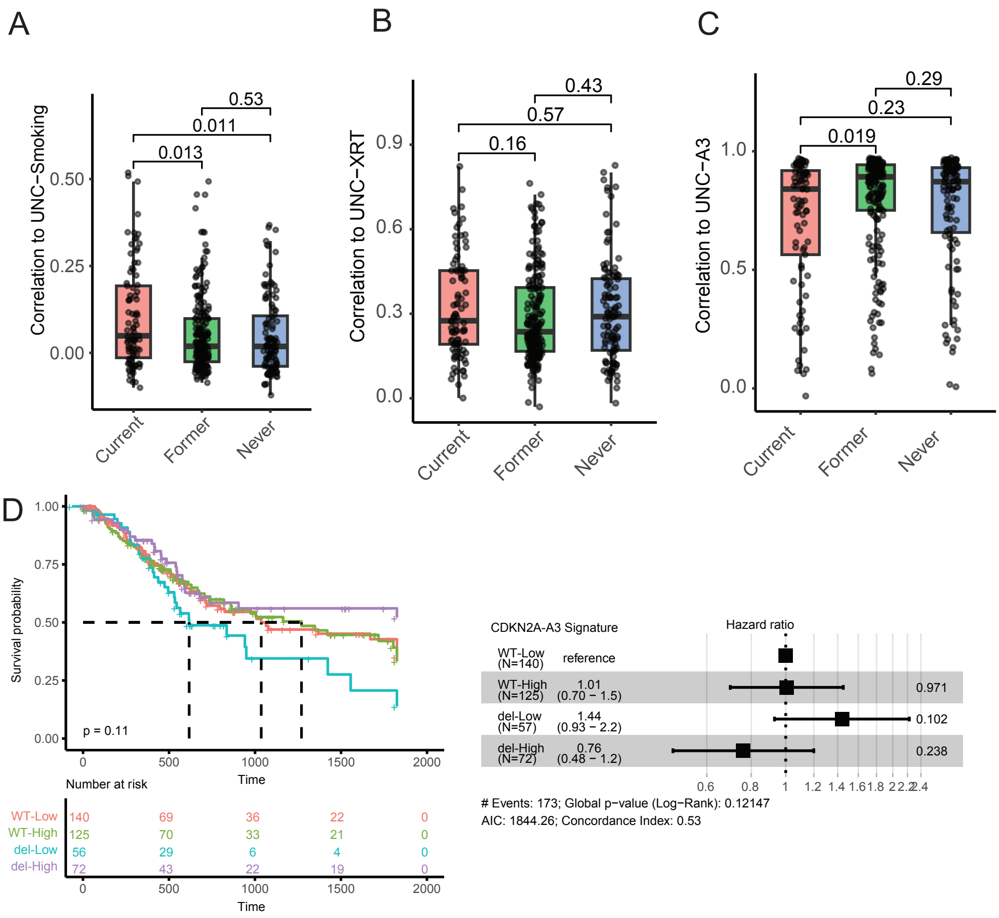

Supplementary Figure S6. Signature validation in TCGA samples. Boxplots displaying correlation between TCGA samples and the **(A)** UNC Smoking, **(B)** XRT, and **(C)** A3-Combo signatures. Samples are grouped by smoking status. Boxplots are represented by the IQR and midline at the median. Error bars equal the Q1/Q3 ± 1.5 × IQR. **(D)** Samples were grouped by high and low signature correlation, relative to the median, and then further subdivided by CKDN2A copy number loss. Overall survival was then visualized by KM plot. Significance was then determined by a cox proportional hazard model.
